# Attributes of sensory and instrumental analysis that determine overall liking of fresh orange varieties with different maturity index

**DOI:** 10.1002/fsn3.4133

**Published:** 2024-04-22

**Authors:** Amparo Baviera‐Puig, Isabel López‐Cortés, María Dolores Ortolá, Juan Buitrago‐Vera

**Affiliations:** ^1^ Department of Economics and Social Sciences Universitat Politècnica de València Valencia Spain; ^2^ Department of Plant Production, COMAV Universitat Politècnica de València Valencia Spain; ^3^ Institute of Food Engineering‐FoodUPV Universitat Politècnica de València Valencia Spain

**Keywords:** Navel, sweet, acidity, juiciness

## Abstract

Spain is the leading exporter of fresh citrus for consumption. In order to adapt to market needs, it is key to analyze the attributes that most influence each variety, the time of ripening and the color of the fruit. In this way, the producer can select the right fruit for each moment of the season. The main objective was to evaluate the fruit quality of three interesting orange tree varieties for fresh consumption: “Washington Navel,” “Navelate,” and “Lanelate” sweet orange. To achieve this goal, sensory analysis was combined with instrumental analysis. In Washington Navel, the maturity index provided a positive contribution to overall liking. Both herbaceous and fruity aroma have a negative influence on the overall liking score. In Navelina, the color and aromas (herbaceous and fruity) played an important positive role in overall liking. The importance of this smell could be related to the freshness of the oranges. In contrast, the maturity index provided a negative contribution. Lanelate is the only variety in which vitamin C provided a positive contribution to overall liking. However, L* value showed a negative contribution. Therefore, there were clearly different characteristics among varieties that were detected. So far, different orange varieties have not been compared according to their maturity index and using sensory analysis. Descriptive analysis and the variables that influence overall liking are fundamental to adapt to consumer expectations. The results obtained would help to select the fruit with better acceptability in the market.

## Graphical abstract

The main objective is to evaluate the fruit quality of three interesting orange tree varieties for fresh consumption. To achieve this objective, sensory analysis is combined with instrumental analysis. The results obtained would help to select the fruit with better acceptability in the market.

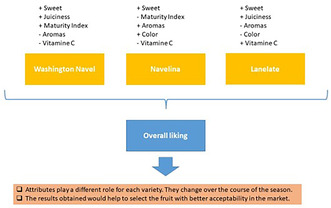

## 1 INTRODUCTION

Spain stands out in citriculture not only for its production but also because it is the leading exporter of fresh fruit for consumption, with almost 60% of its harvest exported to the world. In 2020, the main exporters of fresh or dried citrus were Spain ($4.16MM), South Africa ($1.84MM), China ($1.33MM), Turkey ($985M), and Egypt ($946 M; FAOSTAT, 2021). This crop is mainly produced in the Regions of Valencia and Andalusia, with 49.31% and 41.45% of the Spanish orange tree area, respectively. The Navel and Valencia varieties are predominant in Spain (Ministry of Agriculture Fisheries and Food, 2021). Their moderate resistance to frost, their adaptability to a wide range of climatic conditions and the diversity of cultivars available make them very adaptable to many regions (Alcon et al., 2019).

In the Valencia region, the usual cultivars are Navel oranges due to their productivity and high‐quality fruits for fresh consumption (Legua et al., 2011). Consumers demand these varieties for their balance of sweetness and acidity and for their antioxidant and antimicrobial properties (Bull et al., 2004; Freeman et al., 2010; Tietel et al., 2011). Throughout the season, the fruit matures and changes in sugar content (Kahn et al., 2007; Tadeo et al., 1987). The relative concentration of the different volatile aromatic compounds also evolves and depends on the maturation stage (Hou et al., 2020). In order to adapt to market needs, analyzing the attributes that most influence each variety, the time of ripening and the color of the fruit is crucial. This way, the grower can select the right fruit for each moment of the season and kind of consumer (Pereira et al., 2017; van Rijswijk & Frewer, 2008).

Therefore, the main aim of this study is to evaluate the fruit quality of three interesting orange varieties for fresh consumption in order to allow the producer (and the rest of the actors in the supply chain) to classify the fruit according to the final destination. The samples were collected throughout the season. The specific objectives of the paper were (1) to determine what attributes characterize each variety of oranges, (2) find out if the same variety is perceived differently depending on its maturity index, (3) define the effect that each attribute has on the overall liking by variety, and (4) analyze how each variety evolves throughout the campaign.

To achieve this objective, sensory analysis was combined with instrumental analysis. Sensory analysis was used to measure the components of taste, touch and aroma, while the physicochemical characteristics were defined with instrumental analysis (López et al., 2007; Murray et al., 2001; Torrico et al., 2018).

## 2 MATERIALS AND METHODS

### 2.1 Plant material

Experiments were carried out over the 2021–2022 campaign in commercial orchards of “Washington Navel,” “Navelate,” and “Lanelate” sweet orange [*Citrus sinensis* (L.) Osb.] located in Valencia (Spain). The trees were 20–25 years old and had been budded onto Carrizo citrange rootstock (*Poncirus Trifoliata* x *C. sinensis*). The trees were planted 6 × 4–5 m apart and grown in a calcareous loamy–silty clay soil, with drip irrigation. The plots were located in the central area of the Valencian Citrus Protected Geographical Indication (Valencia, Spain 39°30′ N, 0°28′ W). Fertilization, pruning and pest management were the same for all samples collected. All varieties were grown in the same way so that this would not influence the final result.

Sampling was carried out in a randomized design among four plots of each cultivar, all of them in the same population and in close proximity. From a total of nine trees of each variety, three replicates of two samples were taken. In the laboratory, two samples were chosen from each of them. Sampling was carried out in three replicates so that the choice of fruit would not be affected by the position of the tree. Sampling was initiated when the commercial standard was met for each variety (minimum size >87–100 mm diameter; minimum juice content >35%; maturity index ≥6.5; coloring >6). Five successive additional samples were taken 15 days apart (Table 1).

**TABLE 1:** Codes and dates of samples harvested.

Washington Navel, Samples	Harvest day	Navel samples	Harvest day	Lanelate samples	Harvest day
WN1	1 Nov.	N1	2 Jan.	L1	1 Feb.
WN2	15 Nov.	N2	15 Jan.	L2	15 Feb.
WN3	1 Dec.	N3	1 Feb.	L3	1 Mar.
WN4	15 Dec.	N4	15 Feb.	L4	15 Mar.
WN5	2 Jan.	N5	1 Mar.	L5	1 Apr.
WN6	15 Jan.	N6	15 Mar.	L6	15 Apr.

### 2.2 Instrumental analysis

To evaluate the possible differences detected by the trained panelists, 10 fruits from each field sampling were analyzed physicochemically with the following parameters:

1. Soluble solids content (SSC) was measured in the juice with a refractometer (Zeiss, ATAGO PR101, Japan) at 20°C, obtaining the results in °Brix.
2. Titratable acidity was determined in the juice by potentiometric titration (905 Titrando, Metrohm) with 0.1 N NaOH of up to pH 8.1–8.2. Results were expressed as g of citric acid/L.
3. Maturity Index (MI) was calculated as SSC/acidity ratio (Lado et al., 2014).
4. Vitamin C content in the three fresh orange juices was determined by potentiometric titration (905 Titrando, Metrohm) with sulfuric acid (25% w/v) with 2, 6 dichlorophenolindophenol (0.05 mol/L by 1:5 dilution with distilled H_2_O). The results were expressed as mg of ascorbic acid/L.
5. Optical properties of peel were determined with a spectrocolorimeter (Konica Minolta, Inc., model CM‐3600d, Tokyo, Japan) using a 10° viewing angle, standard illuminant D65 and the CIELab color space. For each fruit, three readings were performed on the equatorial zone, obtaining an average of the three readings (L*, a* and b*). The a* colorimetric coordinate corresponded to the green‐red axis, where the negative values were related to the green color and the positive values with the orange and red (−60 green, +60 red). The b* corresponded to the blue‐yellow axis, where the negative values were related to the color blue and the positive values with yellow (−60 blue, +60 yellow). The L* coordinate (0 = black, 100 = white) measured the luminosity and was placed on the z‐axis, with the a* and b* coordinates placed on the xy color planes (Ben Abdelaali et al., 2018). Chroma (C* = (a*)^2^ + (b*)^2^)^1/2^) is the quantitative colorfulness attribute, as it determines the difference degree in comparison to a gray color with the same lightness for each hue. Hue angle (h* = 1/tan(b*/a*) is a parameter that defines the colors traditionally as pinkish, yellowish and greenish (Meléndez‐Martínez et al., 2003; Weatherall & Coombs, 1992).

Color index (CI) was calculated as

$$\text{Peel}\;\mathrm{CI}=\frac{1000\cdot a^{*}}{L^{*}\cdot b^{*}}$$

### 2.3 Sensory analysis

For sensory analysis, the fruit samples were kept under controlled conditions. The tasting panel consisted of tasters trained over the last 5 years, in which training sessions were conducted for the recognition and rating of the characteristic visual and tactile attributes of the fruit (Bettini et al., 1998). All panelists were regular orange consumers. The panel consisted of 16 judges (8 women and 8 men), aged 25 to 70 years. Between 8 and 12 tasters are required for each test, although it is wise to keep some extra tasters in reserve to cover possible absences (COI/T.20).

In the training sessions, olfactory and taste tests were performed with prepared artificial aromas (Le nez du vin. Jean Lenoir). These were always aromas related to the fruit to be tasted, and taste tests that prepared different scales of acidity and sweetness, among others. The training sessions were held weekly.

The tastings took place in a suitable room which, in addition to being odor‐free, must be sufficiently insulated and comfortable, and must be under controlled ambient temperature as mentioned above. The evaluation was conducted from 10:00 a.m. to 1:00 p.m. in individual booths with controlled lighting and temperature. At each tasting, two oranges for each of the samples were placed on white plates and presented to the panel. The fruits were removed from cold storage, equilibrated to room temperature and placed as random samples of whole, intact fruit for tasting by the panelists. Each sample was identified by a random three‐digit code, and all samples were of uniform size. The order of the samples for each panelist was randomized, and they were provided with water and invited to drink after each sample tasted (Baxter et al., 2005). Depending on the harvest dates, one or two different varieties were tasted. When more than one variety was tested, the taster had a repeated sample as a control system for the taster himself/herself.

The tasting session was carried out by means of the tasting sheet, which made it possible to establish the sensations conveyed by the fruit during the sensory analysis (Baxter et al., 2005). Tasters were also provided with the material necessary to be able to peel and cut the fruit. Each tasting session was divided into four different parts, in which the visual, tactile, gustatory and olfactory phases were evaluated (Chen & Nussinovitch, 2001). In each of the phases, an evaluation was made in order to have a complete characterization of the orange (Lotong et al., 2003).

Using a colorimetric scale (Pointer & Attridge, 1997) that we have adapted to the colors of citrus fruits from the Mediterranean climate zone, we studied the color of both the peel and the flesh of the samples. In the taste phase of orange, it is interesting to study the balance between sugar and acidity, on which the assessment of bitter and astringent taste depends, as reported in the article by Baxter et al. (2005), in order to have joint knowledge of the parameters.

To prepare for the sensory evaluation, the panelists, in preliminary sessions using commercial and experimental fruit samples, generated the sensory descriptors in the final sensory test. They were asked to score each sample using the tasting sheet developed. The panelist evaluated the samples using a category scale, assigning each descriptor a score from 1 (low intensity of the attribute) to 9 (high intensity of the attribute). The attributes were ranked as follows:

1. Touch: peel roughness, peel color, flesh color (in the whole fruit), ease of peeling and presence of seeds.
2. Odor: herbaceous and fruity aroma in the fruit segments.
3. Flavor: sweetness, acidity, bitterness and juiciness.
4. Overall liking.

In the meeting that took place at the end of each tasting session, the tasters presented their scores, opinions and points of view, without modifying in any case the data that had been noted on the tasting sheet. The consensus was obtained from the report on each of the samples.

This study was approved by the Research Ethics Committee of the Universitat Politècnica de València (UPV), and informed consent was obtained from each subject prior to their participation in the study.

### 2.4 Statistical analysis

All samples were characterized by their average measurement (instrumental analysis) and their average score among all panelists (sensory evaluation). The average values of the soluble solids, acidity and vitamin C analyses were calculated with the 10 replicates of each field sampling, while the optical properties, for which three measurements were taken from each fruit, were averaged over 30 analyses. Before calculating the average score for all panelists, scores not exhibiting a normal distribution were removed. All data were tested by analysis of variance (ANOVA) for every variety. Means were separated by Duncan's Multiple Range Test (MRT) at *p* ≤ .05. Pearson's correlation coefficients were calculated among all variables. Significant correlation coefficients (*p* < .005) were considered moderate when .55 < |*r*| < .70 and strong when |*r*| ≥ .70. Data were normalized using the inverse of the standard deviation of each variable (1/SD) to avoid dependence on measured units (Martens & Naes, 1992).

Then, multivariate analysis was carried out. A Principal Component Analysis (PCA) was performed involving all the samples and variables considered. After this, partial least‐square regression (PLSR) was used to quantify the correlation between all the variables (instrumental and sensory, *X* data) and overall liking (*Y* data) for every variety (de Oliveira Rocha & Bolini, 2015; Liu et al., 2015; Toscas et al., 1999).

To facilitate interpretation of the results and to identify variables providing higher contribution to the model, some variables were removed. The importance of the explanatory variables for the building of the *t* components was deduced by the variable importance for the projection (VIPs), which allows identifying the variables that are moderately (0.8 < VIP < 1) or highly influential (VIP ≥ 1). A further modeling with PLSR was explored using the selected variables based on VIP values (Piombino et al., 2013). All analyses were conducted using XLSTAT (Addinsoft; Vidal et al., 2020).

## 3 RESULTS AND DISCUSSION

### 3.1 Description of the samples

First, the samples for every variety are described. The ANOVA revealed significant differences among them. First, in Washington Navel variety (Table 2), there were significant differences ins overall liking, easy peeling, sweetness and bitterness. The best rated sample for overall liking was WN6 (6.86). The easiest samples to peel were WN1 (7.20), WN3 (6.80), WN4 (7.20) and WN6 (7.07), the sweetest were WN3 (6.00), WN4 (6.45), WN5 (6.00) and WN6 (5.93), and the most bitter WN1 (1.40) and WN2 (1.42). All samples had a maturity index above 6.5, so they met the quality standards required for export (EU, 2011).

**TABLE 2:** Mean sensory and instrumental scores for Washington Navel samples (WN1–WN6).

Sample		WN1	WN2	WN3	WN4	WN5	WN6	Pr > *F*(sample)
Overall liking		5.65^a^	5.84^a^	6.25^a^	6.20^a^	6.00^a^	6.86^b^	.002
Touch	Peel roughness	5.3	5.21	6	5.2	4.64	4.93	.356
	Peel color	18.15	18.16	18	18.75	18.5	18.14	.151
	Pulp color	15.65	15.53	15.85	15.7	15.71	15.64	.986
	Easy peeling	7.20^b^	6.37^ab^	6.80^b^	7.20^b^	5.64^a^	7.07^b^	.017
Smell	Herbaceous aroma	2.2	2	1.45	2.05	1.36	1.5	.451
	Fruity aroma	5.1	5.32	5.35	6.05	6.43	5.71	.428
Taste	Sweet	5.05^a^	5.11^a^	6.00^b^	6.45^b^	6.00^b^	5.93^b^	.000
	Acidity	5.5	5.05	5.2	4.7	4.36	5.07	.376
	Bitter	1.40^b^	1.42^b^	1.05^a^	1.15^ab^	1.00^a^	1.07^ab^	.044
	Juiciness	6.5	6	6.9	6.95	6.36	7	.122
Instrumental analysis	SSC (°Brix)	10.77^ab^	11.01^b^	10.32^a^	10.92^ab^	12.94^c^	11.84^bc^	<.0001
	Acidity (g/L)	10.72^b^	9.16^a^	11.07^b^	8.94^a^	11.81^b^	9.8^a^	<.0001
	Maturity Index	10.11^b^	12.33^c^	9.32^a^	12.38^c^	10.98^b^	12.15^c^	<.0001
	L*	65.86^c^	65.76^bc^	67.79^d^	64.51^b^	62.22^a^	65.99^cd^	<.0001
	a*	38.75^b^	39.85^b^	35.18^a^	39.64^b^	39.34^b^	38.31^a^	<.0001
	b*	65.42^b^	64.97^b^	67.77^c^	61.59^a^	60.25^a^	63.86^b^	<.0001
	C*	76.14^c^	76.28^c^	76.41^c^	73.28^a^	72^a^	74.57^b^	<.0001
	h*	59.31^bc^	58.46^ab^	62.57^d^	57.21^a^	56.81^a^	58.98^c^	<.0001
	Peel CI	9.09^b^	9.39^bc^	7.68^a^	10.03^cd^	10.57^d^	9.18^b^	<.0001
	Vitamin C (mg/g)	0.61	0.6	0.61	0.58	0.61	0.55	.3811

*Note*: Values with different letters are significantly different at *p* ≤ .05 Duncan's MRT.

As for the physicochemical parameters, although statistically significant differences were found between the samples in relation to their SSC, these differences did not seem to be detected by the tasters. Thus, sample WN5, with the highest sugar content, was not detected as the sweetest. In fact, tasters did not differentiate WN3, WN4, WN5 and WN6 samples by sweetness. Probably, they were not able to distinguish the different levels of soluble solids (mostly sugars) among the samples, or the level of sweetness they indicated may have been the aggregate of several sensory attributes, such as the level of acidity and aroma (Obenland, Collin, Sievert, et al., 2009). The same occurred with the acid content.

Furthermore, the tasters did not differentiate the color of the peel according to the color coordinates analyzed. The WN4 and WN5 samples, with a higher CI as a result of their higher value in the a* coordinate, were those that obtained the highest score according to the colorimetric scale (Pointer & Attridge, 1997). In this scale, 18 and above is considered a suitable color for Washington Navel.

Table 3 describes the Navelina samples. The ANOVA revealed significant differences in overall liking, peel color, pulp color, easy peeling, sweetness, acidity and bitterness. The best rated sample for overall liking was N5 (5.74). The easiest sample to peel was N3 (6.31), and the sweetest was N6 (7.20).

**TABLE 3:** Mean sensory and instrumental scores for Navelina samples (N1–N6).

Sample		N1	N2	N3	N4	N5	N6	Pr > *F* (sample)
Overall liking		4.75^a^	4.94^a^	5.00^a^	5.69^ab^	5.74^b^	5.60^ab^	.024
Touch	Peel roughness	4.58	4.78	4.25	4.81	3.7	4.52	.076
	Peel color	17.67^ab^	18.34^b^	17.44^a^	18.19^b^	18.11^b^	17.00^a^	.001
	Pulp color	11.00^a^	12.69^ab^	13.88^bc^	13.63^bc^	15.00^c^	13.00^ab^	.002
	Easy peeling	5.08^ab^	4.75^a^	6.31^c^	6.06^bc^	5.59^b^	4.20^a^	<.0001
Smell	Herbaceous aroma	3.42	3.59	2.94	3.19	3.41	2.8	.686
	Fruity aroma	5.58	5	5.94	5.88	5.96	7	.073
Taste	Sweet	4.08^a^	4.22^a^	4.63^a^	5.50^b^	5.81^b^	7.20^c^	<.0001
	Acidity	5.75^bc^	5.63^bc^	5.94^c^	4.69^b^	5.33^bc^	2.20^a^	<.0001
	Bitter	1.42^ab^	1.28^ab^	1.50^ab^	1.31^ab^	2.26^b^	1.00^a^	.031
	Juiciness	6	6.06	6.19	6.38	6.22	4.8	.252
Instrumental analysis	SSC (°Brix)	9.75^a^	9.76^a^	10.62^b^	10.66^b^	11.06^c^	11.36^d^	<.0001
	Acidity (g/L)	8.01^a^	8.06^a^	11.28^b^	11.09^b^	11.2^b^	12.02^c^	<.0001
	Maturity Index	12.32^c^	12.25^c^	9.49^a^	9.68^a^	9.92^b^	9.49^a^	<.0001
	L*	67.57^bc^	67.24^bc^	69.87^c^	69.64^c^	66.66^b^	65.16^a^	<.0001
	a*	29.91^a^	30.36^a^	33.92^b^	33.97^b^	37.38^c^	39.85^d^	<.0001
	b*	44.51^b^	43.92^a^	67.45^d^	67.25^d^	64.45^c^	63.34^c^	<.0001
	C*	53.73^a^	53.51^a^	75.67^b^	75.5^b^	74.64^b^	74.93^b^	<.0001
	h*	56.05^a^	55.27^a^	63.28^c^	63.16^c^	59.87^b^	57.85^a^	<.0001
	Peel CI	10.06^c^	10.44^c^	7.33^a^	7.38^a^	8.81^b^	9.71^c^	<.0001
	Vitamin C (mg/g)	0.55^bc^	0.56^c^	0.52^ab^	0.56^c^	0.49^a^	0.49^a^	.0001

*Note*: Values with different letters are significantly different at *p* ≤ .05 Duncan's MRT.

In this variety, the significant differences observed in the samples in relation to SSC were detected by the tasters, who found the N6 sample to be sweeter, as it contained a higher amount of sugars. As for acidity, tasters considered samples N1, N2, N3 and N5 to be the most acidic, which does not correspond to the acidity analyzed, where sample N6 had the highest titratable acid content. This would be related to the sugars/acidity ratio, since the tartness of acids is reduced by sugars (Tyl & Sadler, 2017). On the other hand, the significant differences found in the color coordinates were not detected by the tasters. Thus, the tasters rated the skin color of samples N2, N4 and N5 higher, while the highest calculated CIs were for samples N1, N2 and N6.

Table 4 describes the Lanelate samples. The ANOVA revealed significant differences in overall liking, easy peeling, sweetness, acidity and juiciness. The best rated sample for overall liking was L2 (6.64). The easiest samples to peel were L3 and L4 (6.60 and 6.50, respectively), and the sweetest was L6 (7.50). In this case, the significant differences observed in the soluble solids content have been detected by the tasters, as L6 is the one with the highest SSC. The most acidic samples were L1 (5.85) and L3 (5.10), samples that had a significantly higher titratable acidity content than the rest of the samples. The juiciest were L1 (6.90) and L2 (6.79).

**TABLE 4:** Mean sensory and instrumental scores for Lanelate samples (L1–L6).

Sample		L1	L2	L3	L4	L5	L6	Pr > *F* (sample)
Overall liking		6.25^bc^	6.64^c^	5.70^b^	6.15^bc^	4.35^a^	6.20^bc^	<.0001
Touch	Peel roughness	5.1	5.93	5.25	5	4.5	4.75	.162
	Peel color	18.05	18	17.85	18.35	18.3	18.3	.136
	Pulp color	17.05	16.79	17.45	16.85	17.4	16.9	.341
	Easy peeling	5.15^a^	6.14^ab^	6.60^b^	6.50^b^	5.45^ab^	6.10^ab^	.044
Smell	Herbaceous aroma	2.7	1.64	1.85	1.85	2.65	2.4	.105
	Fruity aroma	5.65	6.5	6.3	6.65	6.25	6.15	.636
Taste	Sweet	5.55 ^ab^	6.71 ^cd^	6.70 ^cd^	6.35^bc^	5.05^a^	7.50^d^	<.0001
	Acidity	5.85^b^	3.79^a^	5.10^b^	3.05^a^	3.70^a^	3.10^a^	<.0001
	Bitter	1.05	1.07	1.3	1	1.2	1.15	.321
	Juiciness	6.90^c^	6.79^c^	6.60^b^	6.65^b^	4.65^a^	6.65^b^	<.0001
Instrumental analysis	SSC (°Brix)	12.33^c^	11.39^b^	12.33^c^	10.64^a^	11.68^b^	14.54^d^	<.0001
	Acidity (g/L)	12.58^e^	6.25^a^	9.28^d^	6.56^ab^	7.27^c^	6.74^b^	<.0001
	Maturity Index	9.89^a^	18.4^d^	13.28^b^	16.26^c^	16.07^c^	21.65^e^	<.0001
	L*	65.96^d^	64.3^bc^	65.41^cd^	63.65^ab^	65.48^cd^	62.61^a^	<.0001
	a*	37.68^ab^	38.47^b^	36.34^a^	38.64^b^	37.72^ab^	36.34^a^	.0044
	b*	65.07^d^	62.44^c^	64.16^cd^	59.35^b^	62.92^c^	55.81^a^	<.0001
	C*	75.28^d^	73.41^c^	73.81^cd^	70.91^b^	73.43^c^	66.65^a^	<.0001
	h*	59.88^bc^	58.32^ab^	60.43^c^	56.86^a^	59.02^bc^	56.84^a^	<.0001
	Peel CI	8.87^ab^	9.66^bc^	8.73^a^	10.33^c^	9.23^ab^	10.48^c^	<.0001
	Vitamin C (mg/g)	0.74^e^	0.50^b^	0.59^d^	0.44^a^	0.49^b^	0.54^c^	<.0001

*Note*: Values with different letters are significantly different at *p* ≤ .05 Duncan's MRT.

### 3.2 Attributes that characterize each variety

**FIGURE 1:**
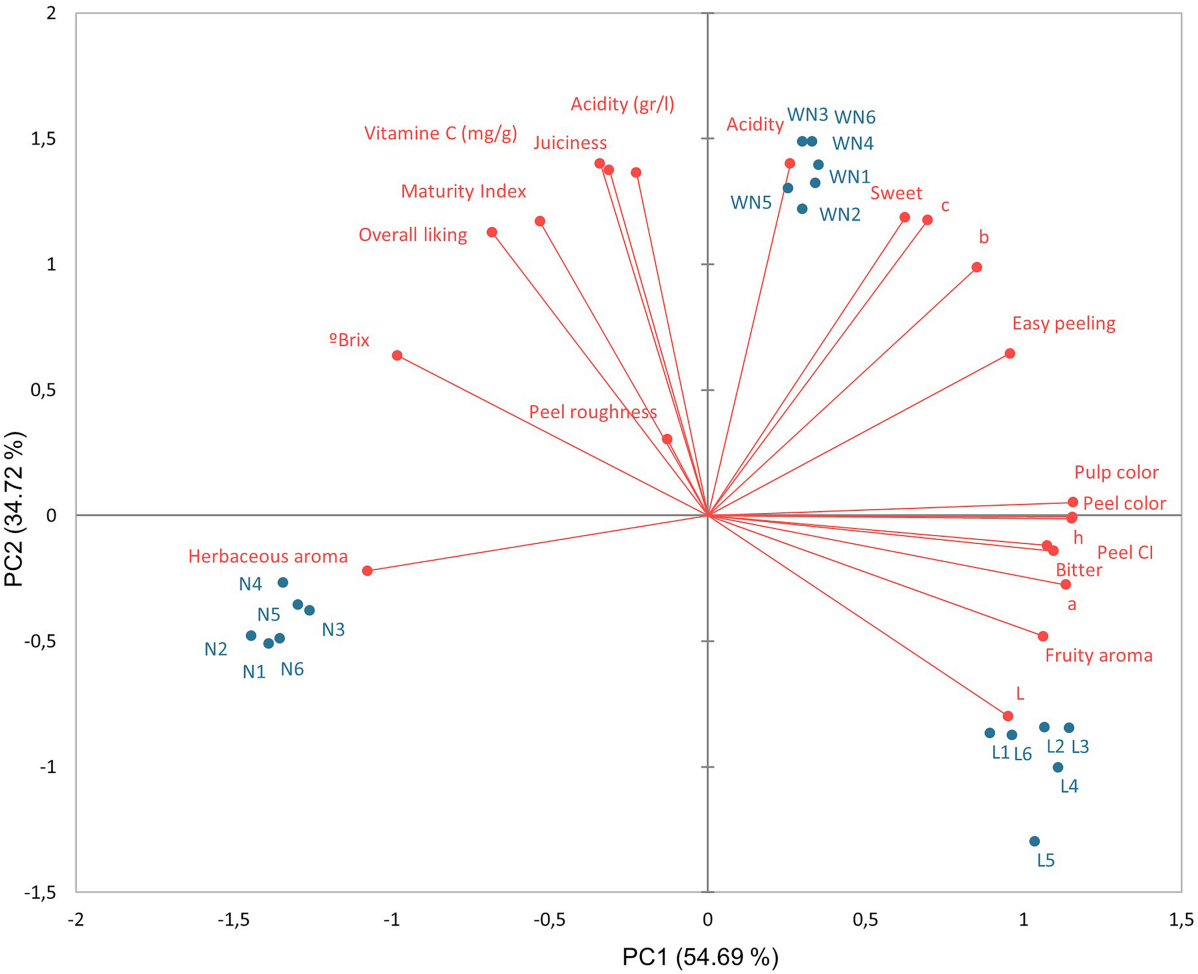
Biplot of PC1 versus PC2 corresponding to a full‐data PCA model for all samples and variables.

A full‐data PCA model was developed to provide a global overview of the different samples and variables (research objective 1). Principal components 1 (PC1) and 2 (PC2) accounted for 54.69% and 34.72% of total variance, respectively. In total, this represented 89.41%. The biplot of PC1 versus PC2 for this PCA model (Figure 1) shows three well‐differentiated groups, as the samples were distributed according to their variety. Washington Navel was related to acidity and sweetness. Navelina was related to herbaceous aroma. Lanelate was linked to fruity aroma. Therefore, it can be seen how each variety was perfectly distinguishable from the others and had different characteristics. Varieties matter, whether consumers realize it or not (Lado et al., 2014; Poole et al., 2007).

### 3.3 Correlations between physicochemical parameters and sensory descriptors

Pearson correlation analysis was conducted for each variety using all the variables (research objective 2). It should be noted that a strong negative correlation was observed in our study between acidity (g/L) and MI for Washington Navel (*r* = −.824), Navelina (*r* = −.938) and Lanelate (*r* = −.855). As Divya et al. (2016) observed, for the same maturity index, the lower the acidity, the higher the quality.

In particular, there was a moderate positive correlation between overall liking and juiciness (*r* = .550) for Washington Navel. In the instrumental analysis, there was a strong negative correlation between a* and b* (*r* = −.706). During maturation, vegetable products change their color owing to chlorophyll degradation (related to de coordinate b*) and to the increase in other pigments such as carotenoids or polyphenols (related to coordinate a*) (Olmo et al., 2000).

For Navelina, there was a moderate positive correlation between overall liking and sweetness (*r* = .692). For the instrumental part, there was a positive correlation between acidity and SSC (*r* = .668), a* (*r* = .580), b* (*r* = .783) and C* (*r* = .839). It was also positive between °Brix and b* (*r* = .691) and C* (*r* = .721). However, there was a negative correlation between MI and b* (*r* = −.677) and c* (*r* = −.728).

For Lanelate, there was a moderate positive correlation between overall liking and sweetness (*r* = .507) and overall liking and juiciness (*r* = .593). Sweetness and juiciness were also correlated moderately in a positive way (*r* = .516). There was a negative correlation between MI and b* (*r* = −.572), C* (*r* = −.623) and vitamin C (*r* = −.636). There was a strong positive correlation observed in our study between acidity (g/L) and vitamin C (*r* = −.882).

### 3.4 Effects of the sensory and instrumental variables on overall liking

The effect (positive or negative) of the variables providing higher contribution to the PLSR model on the orange liking was identified (Table 5; research objective 3). The variables that were repeated for the three orange varieties were pulp color, herbaceous aroma, sweetness and vitamin C. The only variable that exerts a positive effect on the three varieties is sweetness. For all of them, the contribution is high. This is consistent with the Pearson's correlations.

**TABLE 5:** Explanatory variables with VIP ≥0.8 and their effect on overall liking.

Variable	Washington Navel	Navelina	Lanelate
Peel roughness			**+0.255**
Peel color		*+0.312*	
Pulp color	*−0.089*	**+0.216**	**−0.219**
Herbaceous aroma	**−0.201**	*+0.180*	**−0.115**
Fruity aroma	*−0.049*	**+0.002**	
Sweet	**+0.100**	**+0.205**	**+0.174**
Acidity		*−0.118*	
Bitter	**−0.145**		**−0.160**
Juiciness	**+0.270**		**+0.352**
SSC (°Brix)		**+0.148**	
Acidity (g/L)		**+0.092**	
Maturity Index	*+0.227*	**−0.076**	
L*			*−0.043*
a*	*−0.114*	**+0.146**	
b*		**+0.099**	
C*		**+0.110**	
h*		*+0.034*	*+0.007*
Peel CI			*+0.008*
Vitamin C (mg/g)	**−0.471**	*−0.035*	*+0.186*
R^2^Y	0.920	0.904	0.992
MSE	0.053	0.080	0.007
RMSE	0.229	0.283	0.082

*Note*: In bold VIP ≥ 1 and in italic 0.8 ≤ VIP < 1.

Pulp color and herbaceous aroma were only positive for Navelina. However, vitamin C (mg/g) was only positive for Lanelate. This latter relationship has been observed by other authors in PLS models analyzing vitamin C content and titratable acidity in Valencia oranges using infrared spectroscopies (Borba et al., 2021).

Only for Navelina, the variables peel color, b* and c* provided a positive contribution to overall liking. The first contribution was small, and the last two were high. Color conditions the acceptability of the product, since it is the first impression perceived by the consumer's eyes (Aslam et al., 2023; Tran et al., 2016). Donadini et al. (2022) found that blond oranges were preferred to red oranges, as is the case in Navelinas. SSC and acidity also provided a positive high contribution to Navelina liking. However, the acidity obtained in the sensorial analysis was negative.

The effect of the bitterness variable obtained in the sensorial analysis was slightly negative for Washington Navel and Lanelate. In contrast, the effect of juiciness for these two varieties was positive. This effect of juiciness was consistent with the Pearson's correlations reported above. In both cases (bitterness and juiciness), the contribution was high.

The h* variable provided a positive small contribution for Navelina and Lanelate. Peel CI was also positive for Lanelate. The a* variable provided a negative contribution for Washington Navel and positive for Navelina (Mokarram et al., 2019).

The fruity aroma variable provided a negative contribution in overall liking for Washington Navel and positive for Navelina. The pattern was the same as for the herbaceous aroma. In the PCA, it can be clearly seen how the herbaceous aroma distinguishes Navelina from the others.

Baviera‐Puig et al. (2021) found that the most important criterion when evaluating the quality of an orange was the fruity smell. These authors suggested that the importance of this smell could be related to the freshness of the oranges. In other words, the more the orange smells, the fresher it looks.

The effect of the MI on the overall liking was positive for Washington Navel, while it was negative for Navelina. Exclusively, the effect of peel roughness on Lanelate liking was positive and the variable L* was negative.

The results of the PLSR model are evaluated by the explanatory power of the model for overall liking (cumulative *R* ^2^ *Y*), the Mean Squared Error (MSE) and the Root Mean Squared Error (RMSE). A good model should have the lower MSE and RMSE and the higher *R* ^2^ *Y* (Liu et al., 2010; Piombino et al., 2013). In the three varieties, the coefficient *R* ^2^ *Y* was greater than 0.9, MSE was lower than 0.08 and RMSE was lower than 0.28.

This study also confirmed that juiciness and sweetness are critical attributes in determining overall liking of Navel oranges, which is in agreement with past studies (Baviera‐Puig et al., 2021; Obenland, Collin, Mackey, et al., 2009; Poole & Baron, 1996; Simons et al., 2018). However, the information for every specific product is important. As can be seen, there are clearly different characteristics among varieties that are detected.

### 3.5 Analysis of the samples throughout the campaign

**FIGURE 2:**
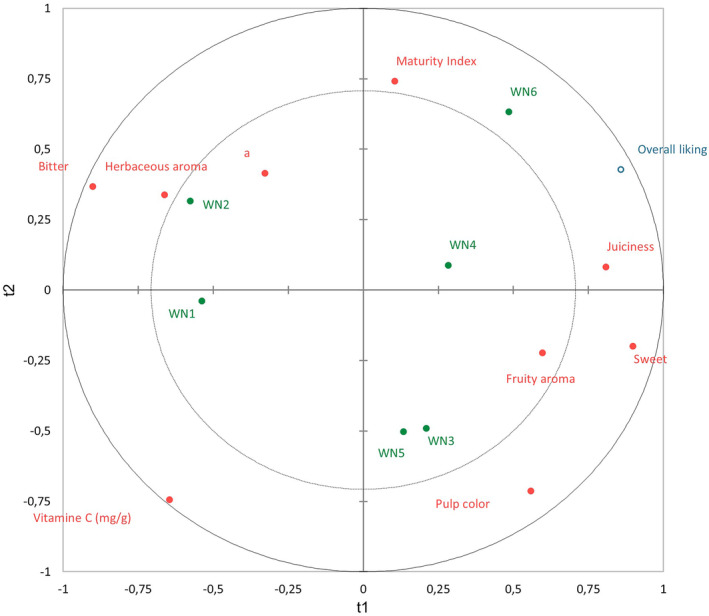
PLS regression for Washington Navel.

**FIGURE 3:**
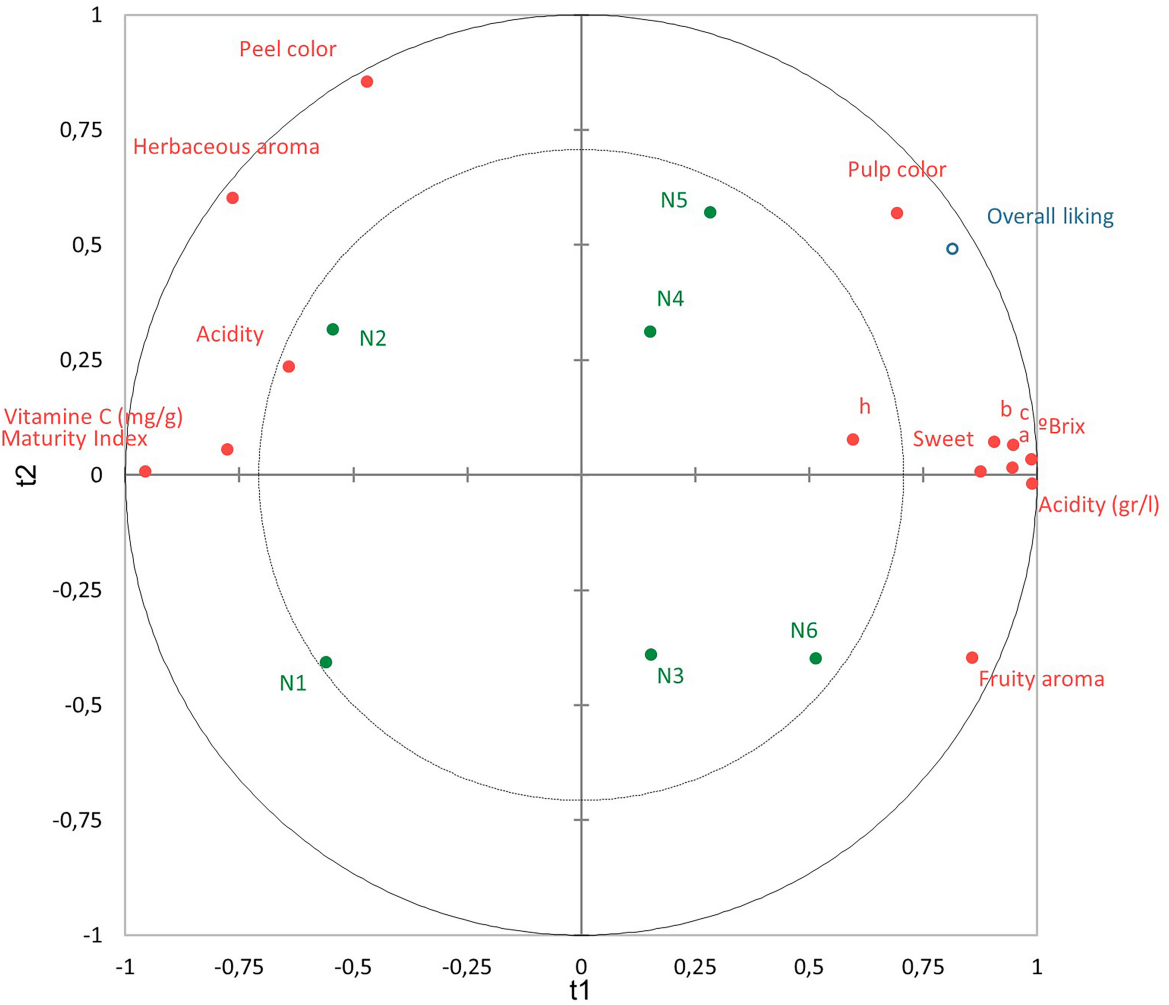
PLS regression for Navelina.

**FIGURE 4:**
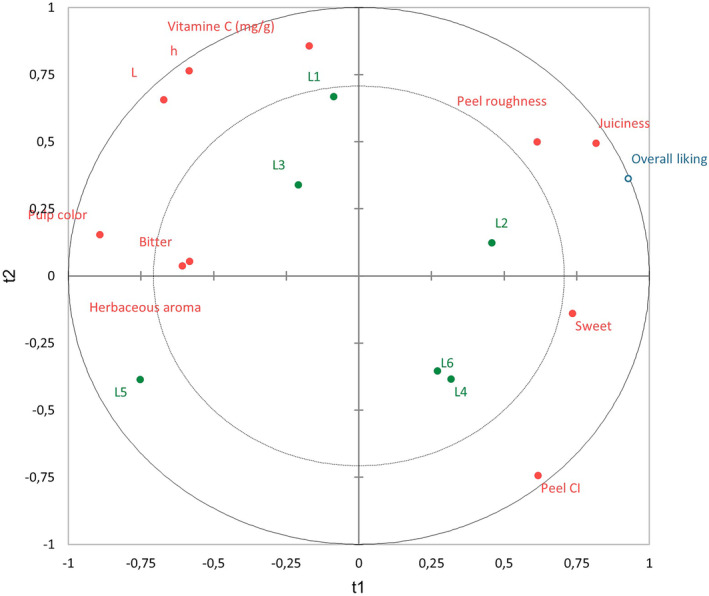
PLS regression for Lanelate.

Once the effect of the different variables was identified, the changes over harvest for each cultivar were analyzed (research objective 4). In Figures 2, 3, 4, objects on the plot that are located nearer to each other are more closely correlated. This analysis compared the sensory and instrumental analyses to the overall liking. The inner and outer circles represent 50 and 100% explained variance, respectively (Frøst et al., 2001).

In Figure 2, it can be observed that the overall liking of the Washington Navel variety was a combination of juiciness and maturity index. In the summary of variables involved in the final evaluation, easy peeling did not appear, due to its known complexity, which was clearly detected in the sensory evaluations. The best rated sample was WN6. WN6 and WN4 are the ones with the highest MI and juiciness. Although their maturity index was similar to WN2, they had a higher sugar and acid content than WN2, which probably made them more pleasant (Poole et al., 2007).

In the case of samples WN3 and WN5, not only sweetness but also pulp color and fruity aroma were valued. They had the highest acid content. Acid was related to the association with the fruity flavor assessed by the panel. WN1 and WN2 stood out for their bitterness and herbaceous components, compared to the rest, which indicated an unsuitable MI for fresh consumption. Their proximity to the variable a* indicated that they were still green/yellow (EU, 2011).

Despite the fact that all the samples were at the optimum harvesting stage, we can see the evolution of the productive season between WN1 and WN5 due to the increase in sugar and fruity aroma.

In Figure 3, the overall liking of Navelina is considered from the pulp color, SSC and sweetness parameters. In the color parameters, the b* component (positive values are yellow colorations) and the chroma or color saturation (the higher the color, the brighter it is) stood out (Donadini et al., 2022). The best rated sample was N5, due to the combination of the cited variables. Among the samples studied, N6 was sweeter. It had a slightly lower sugar content than N4 and N5, but a higher acid content, so its MI was lower.

N1 and N2 were less sweet and less acidic samples, so their MI was the highest. This balance is not typical for the beginning of the season. However, when these oranges were harvested, there was a period of heavy rains which made acidity increase (Roger‐Amat, 1988). Nevertheless, they were the worst rated in overall liking. These samples were the best positioned in terms of vitamin C, as expected, since they were the least ripe samples. Vitamin C contents in different fruits are known to be modulated during fruit development (Mditshwa et al., 2017). N2 also stood out in herbaceous aroma and peel color.

In Figure 4, the overall liking for Lanelate was influenced by many variables, mainly juiciness, peel roughness and sweetness. L2 was the sample with the highest overall liking score and the second highest in juiciness and maturity index. L1 clearly stands out for its vitamin C and, therefore, for its acidity. Chemically, it has the highest acid content (Borba et al., 2021). Perhaps peel roughness appears because it has a greater number of glands and of smaller size that can be confused with roughness. L1 and L3 were samples with higher acidity than the rest of the samples.

Only L6 had a higher sugar content than the rest. L4 and L6, despite being more than 15 days apart, were the same sample in terms of their color index. The CI is higher (more intense orange tones), although L5 also had a CI of the same order. L6 had the highest maturity index. L5 was clearly distinguishable from the rest because of its bitterness and pulp color, indicating that it was not at its optimal time for commercial harvesting. In addition, it had herbaceous aromas.

Studying six samples makes it possible to see the evolution throughout the campaign. In the case of Lanelate, an increase in sugar and a reduction in herbaceous aroma were observed. As Obenland et al. (2008) found, both commercial packing and storage caused reductions in the flavor quality of Navel. Therefore, the results of the study would help to assess the key attributes to monitor this quality throughout the campaign and the supply chain.

However, as one of the limitations of the study, it could be considered that only six samples were collected throughout the season. In future research, a greater number of samples could be collected and analyzed throughout the season to better monitor the evolution of the variables of each variety.

## 4 CONCLUSION

The main aim of this study was to evaluate the fruit quality of three interesting orange varieties for fresh consumption in order to allow the producer (and other supply chain stakeholders) to classify the fruit according to its final destination. First, the attributes that characterized each variety of oranges were determined. Washington Navel was related to acidity and sweetness. Navelina was related to herbaceous aroma, and Lanelate was linked to fruity aroma. Second, there was a strong negative association between acidity (g/L) and maturity index in the three varieties studied. However, there was no correlation between MI and the variables of the sensory analysis. So, the same variety was not perceived differently depending on its maturity index.

Third, the only variable that exerted a positive effect on overall liking for the three varieties is sweetness. For each variety, the maturity index, aromas and color played a different role. In Washington Navel, the MI provided a positive contribution to overall liking. Both herbaceous and fruity aroma had a negative influence on the overall liking score. In Navelina, the color and aromas (herbaceous and fruity) played an important positive role in overall liking. The importance of this smell could be related to the freshness of the oranges. Instead, the MI provided a negative contribution. Lanelate was the only variety for which vitamin C made a positive contribution to overall liking. However, L* value showed a negative contribution.

Fourth, the evolution of each variety throughout the campaign was observed. Washington Navel increased in sugar and fruity aroma. Navelina also increased in sugar, but its content of vitamin C decreased. For Lanelate, an increase in sugar and a reduction in herbaceous aroma was observed.

Descriptive analysis and the variables that influence overall liking are fundamental to be able to adapt to consumer expectations. The results obtained would help select the fruit with better acceptability in the market for each type of consumer. In addition, the consumer can also be assisted at the point of sale by accompanying the product with labels containing information on the key variables obtained, specifically juiciness and sweetness. These variables can give the consumer an overall assessment of the fruit so that they can purchase the one that most suits their preference. The information for every specific product is important, as there are clearly different characteristics among varieties.

## AUTHOR CONTRIBUTIONS

**Amparo Baviera‐Puig:** Formal analysis (equal); funding acquisition (equal); software (lead); writing – review and editing (equal). **Isabel López‐Cortés:** Conceptualization (equal); investigation (equal); methodology (lead); writing – review and editing (equal). **María Dolores Ortolá:** Funding acquisition (equal); methodology (equal); project administration (lead); writing – review and editing (equal). **Juan Buitrago‐Vera:** Conceptualization (equal); methodology (equal); visualization (equal); writing – original draft (lead).

## CONFLICT OF INTEREST STATEMENT

We confirm that there are no relevant financial or non‐financial competing interests to report.

## Data Availability

Data available on request from the authors.
